# BCI-215, a Dual-Specificity Phosphatase Inhibitor, Reduces UVB-Induced Pigmentation in Human Skin by Activating Mitogen-Activated Protein Kinase Pathways

**DOI:** 10.3390/molecules27175449

**Published:** 2022-08-25

**Authors:** Jeong Hyeon Lee, Myoung Eun Choi, Hongchan An, Ju Won Moon, Hye Jin Yeo, Youngsup Song, Sung Eun Chang

**Affiliations:** 1 Department of Biomedical Sciences, Bio-Medical Institute of Technology (BMIT), University of Ulsan College of Medicine, Ulsan 44610, Korea; 2 Department of Dermatology, Asan Medical Institute of Convergence Science and Technology, Asan Medical Center, University of Ulsan College of Medicine, Seoul 05505, Korea; 3 New Drug Development Center, Daegu-Gyeongbuk Medical Innovation Foundation, Daegu 41061, Korea; 4 Department of Biomedical Sciences, University of Ulsan College of Medicine, Asan Institute for Life Sciences, Asan Medical Center, Seoul 05505, Korea

**Keywords:** BCI-215, dual-specificity phosphatase, melanogenesis, mitogen-activated protein kinase, ultraviolet radiation

## Abstract

Background: The dysregulation of melanin production causes skin-disfiguring ultraviolet (UV)-associated hyperpigmented spots. Previously, we found that the activation of c-Jun N-terminal kinase (JNK), a mitogen-activated protein kinase (MAPK), inhibited melanogenesis. Methods: We selected BCI-215 as it may modify MAPK expression via a known function of a dual-specificity phosphatase (DUSP) 1/6 inhibitor. B16F10 melanoma cells, Mel-ab cells, human melanocytes, and a coculture were used to assess the anti-melanogenic activity of BCI-215. The molecular mechanisms were deciphered by assaying the melanin content and cellular tyrosinase activity via immunoblotting and RT-PCR. Results: BCI-215 was found to suppress basal and cAMP-stimulated melanin production and cellular tyrosinase activity in vitro through the downregulation of microphthalmia-associated transcription factor (MITF) protein and its downstream enzymes. The reduction in MITF expression caused by BCI-215 was found to be due to all three types of MAPK activation, including extracellular signal-regulated kinase (ERK), JNK, and p38. The degree of activation was greater in ERK. A phosphorylation of the β-catenin pathway was also demonstrated. The melanin index, expression of MITF, and downstream enzymes were well-reduced in UVB-irradiated ex vivo human skin by BCI-215. Conclusions: As BCI-215 potently inhibits UV-stimulated melanogenesis, small molecules of DUSP-related signaling modulators may provide therapeutic benefits against pigmentation disorders.

## 1. Introduction

Melanin determines the skin color in humans and a dysregulation of melanin synthesis can result in common pigmentary disorders. Melanogenesis is a critical self-defense mechanism against ultraviolet radiation (UV)-induced skin damage and carcinogenesis. However, a dysregulation in the production and distribution of melanin causes skin-disfiguring UV-associated hyperpigmented spots. Melasma, solar lentigines, and post-inflammatory hyperpigmentation (PIH) are typical examples of the effects of overproduced melanin and have caused significant cosmetic problems for millions of people. Several whitening agents have been developed, but their general application has been limited due to their instability and irritability as well as safety concerns. Moreover, the effects of the individual ingredients of currently available products of this nature remain problematic despite numerous testings of different compounds [1,2]. Hence, the development of a whitening agent that is effective and safe is still an unmet need as part of an efficient and affordable treatment regimen for hyperpigmentation [3].

UVB mainly induces the cyclic adenosine monophosphate (cAMP) pathway in melanogenesis. Stem cell factor (SCF) plus endothelin-1 (ET-1) are critical melanogenic factors of UVB-irradiated human skin [4,5]. The activation of the cAMP response element-binding protein (CREB) transcription factor by cAMP induces the expression of the master melanogenic regulator microphthalmia-associated transcription factor (MITF), which is essential for UV-stimulated melanogenesis [6,7].

Melanin synthesis (melanogenesis) occurs in specialized organelles called melanosomes where tyrosinase functions are the most important and rate-limit the enzymes. The transcription of MITF is the central and master step in the regulation of tyrosinase as well as tyrosinase-related protein 1 (Tyrp1) and 2 (dopachrome tautomerase, DCT) [8]. Several mitogen-activated protein kinase (MAPK) signaling pathways are involved in the regulation of MITF expression; one of the most important is the extracellular signal-regulated kinase (ERK) pathway [9]. When ERK is activated to become phosphor-ERK, it phosphorylates MITF at serine 73, thereby degrading it [10]. Hence, a sustained ERK activation leads to a downregulated protein expression of MITF and thus a decrease in tyrosinase and the subsequent melanin synthesis [10,11].

In our previous study, we reported that the activation of MAPK pathways, especially c-Jun N-terminal kinase (JNK) by Ro31-8220, inhibited the CREB-mediated transcription of MITF and subsequent melanin production as well as UV-induced melanogenesis in skin [12]. Ro31-8220 was further discussed to inhibit dual-specificity phosphatase 1 (DUSP1) and the inhibition of DUSP1 enhanced JNK1 activity [12]. Based on this, we hypothesized that the modulation of MAPK by DUSPs may effectively regulate skin pigmentation. DUSPs, protein tyrosine phosphatases that can dephosphorylate tyrosine or serine/threonine residues, are known to inactivate MAPKs, thereby acting as a dominant-negative regulator of MAPK signaling [13,14]. 

**Figure 1:**
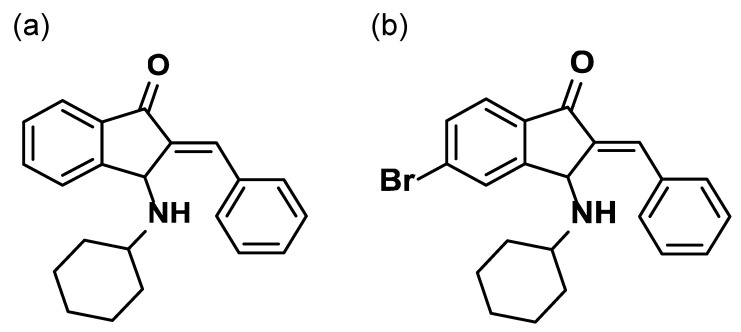
Chemical structure of BCI-215. Chemical structure of (**a**) BCI (CAS#: 1245792-51-1) and (**b**) BCI-215 (CAS#: 1245792-67-9).

After searching for candidate small molecules that could penetrate the skin and modulate MAPK pathways via DUSPs in melanocytes, we found BCI-215, a commercially available chemical. BCI-215 (Figure 1b) is a chemical produced from BCI (Figure 1a) and known as a DUSP1/6 inhibitor. First, we confirmed the anti-melanogenic effect of BCI-215 in B16F10 melanoma cells, which are usually used for anti-melanogenesis screening. We then thought that BCI-215 may regulate melanin biosynthesis via MAPK modulation and could be a candidate for a pigment modulator. In our present study, we investigated its depigmenting effect and working mechanisms in relation to MAPK signaling pathways. 

## 2. Results

### 2.1. BCI-215 Suppresses Melanin Content, Which Was Increased by cAMP 

**Figure 2:**
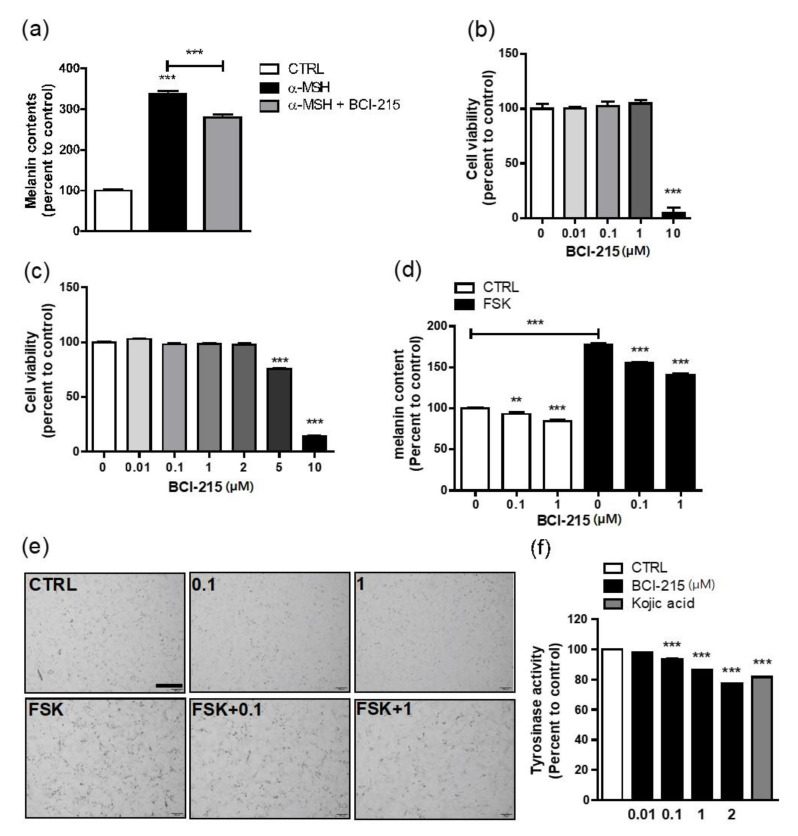
Cellular viability and the effect of BCI-215 on the melanin content and cellular tyrosinase activity. (**a**) Anti-melanogenesis screening. Effect of 1 µM BCI-215 on the cellular melanin content of B16F10 cells stimulated with 100 ng/mL α-MSH for 72 h. Cellular viability was measured using a WST assay in B1610F cells (**b**) and Mel-ab cells (**c**) treated with 0.1–10 µM BCI-215. (**d**) Effect of BCI-215 on melanin content in Mel-ab cells in the conditions of basal or forskolin (FSK) stimulation for 72 h. (**e**) Photographs of cells showing the effects of 0.1 and 1 µM BCI-215 on melanin content of Mel-ab cells in the conditions of basal or FSK stimulation for 72 h. (**f**) Effect of BCI-215 on cellular tyrosinase activity when Mel-ab cells were basally treated with 0.1, 1, and 2 µM BCI-215 for 72 h compared with 200 µM of a well-known whitening agent, kojic acid. ** *p* < 0.01, *** *p* < 0.001 vs. controls.

α-MSH-stimulated B16F10 melanoma cells were used to screen the anti-melanogenic effects of the candidate molecules. A treatment of 1 µM BCI-215 for 72 h following a pre-treatment with 100 nM α-MSH statistically significantly decreased the melanin content in the B16F10 cells (Figure 2a). The cytotoxicity of the BCI-215 exposure in the B16F10 cells was assessed using a WST cell proliferation assay and 10 µM BCI-215 showed cytotoxic effects (Figure 2b). Having observed the anti-melanogenic effect of BCI-215 in the B16F10 cells, we subsequently investigated the effect of BCI-215 on melanogenesis in more appropriate Mel-ab melanocytes because B16F10 cells are melanoma cells. When the cell viability was measured by a WST cell proliferation assay in the Mel-ab cells, BCI-215 at 5 µM started to show cytotoxic effects (Figure 2c). As UV-associated pigmentation involves the cAMP-stimulating pathway in melanogenesis, we stimulated Mel-ab cells with forskolin (FSK), which is a known cell-permeable adenylyl cyclase agonist. The FSK treatment increased the melanin content whereas exposure to 0.1 µM and 1 µM BCI-215 for 72 h significantly reduced the melanogenic effects of FSK (Figure 2d,e). A 1 µM dose of BCI-215 for 72 h produced a basally modest reduction in the melanin content of the Mel-ab cells. BCI-215 reduced the melanin content to a greater extent when the cells were stimulated with FSK, which was evident in the photographs (Figure 2d,e). Furthermore, BCI-215 suppressed cellular tyrosinase activity in a dose-dependent manner and more potently compared with a well-known whitening agent, kojic acid (Figure 2f). These results suggested that BCI-215 profoundly inhibited the melanin content due to the production of a smaller amount of tyrosinase protein from the Mel-ab cells.

### 2.2. BCI-215 Does Not Downregulate the Transcription of MITF and Its Downstream Melanogenic Genes in a 24 h Frame 

**Figure 3:**
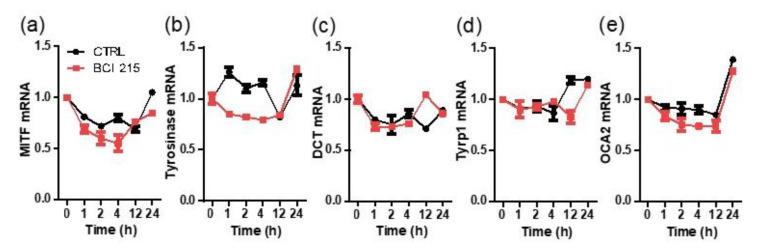
Effect of BCI-215 on the transcription of *MITF* and its downstream genes, *tyrosinase, Tyrp1, DCT*, and *OCA2*. Time-dependent curve of the mRNA expression levels in Mel-ab cells treated with 1 µM BCI-215 for up to 24 h. (**a**) Relative value of *MITF* mRNA. BCI-215 decreased the transcription of MITF modestly only at an early time point. (**b**–**e**) Relative value of *MITF* target genes, including *tyrosinase, Tyrp1, DCT*, and *OCA2*. The control mRNA curve is shown in each graph.

We then determined whether BCI-215 affected the transcription of *MITF*, which is a master downstream regulator of tyrosinase and other melanogenic genes in Mel-ab cells. BCI-215 was used at a 1 µM concentration for the following experiments. When we quantified the relative mRNA expression of *MITF* up to 24 h after exposure to BCI-215, only a marginal decrease in transcription was observed at the early time point and was otherwise unchanged. Both the control and treated samples showed a natural fluctuation in *MITF* transcription in the melanocytes in vitro (Figure 3a). As expected, the target downstream genes of *MITF* such as *tyrosinase, DCT, Tyrp1*, and *OCA2* were not decreased by BCI-215 compared with the control in a 24 h frame (Figure 3b–e). 

### 2.3. BCI-215 Downregulates the MITF Protein and Downstream Melanogenic Proteins 

**Figure 4:**
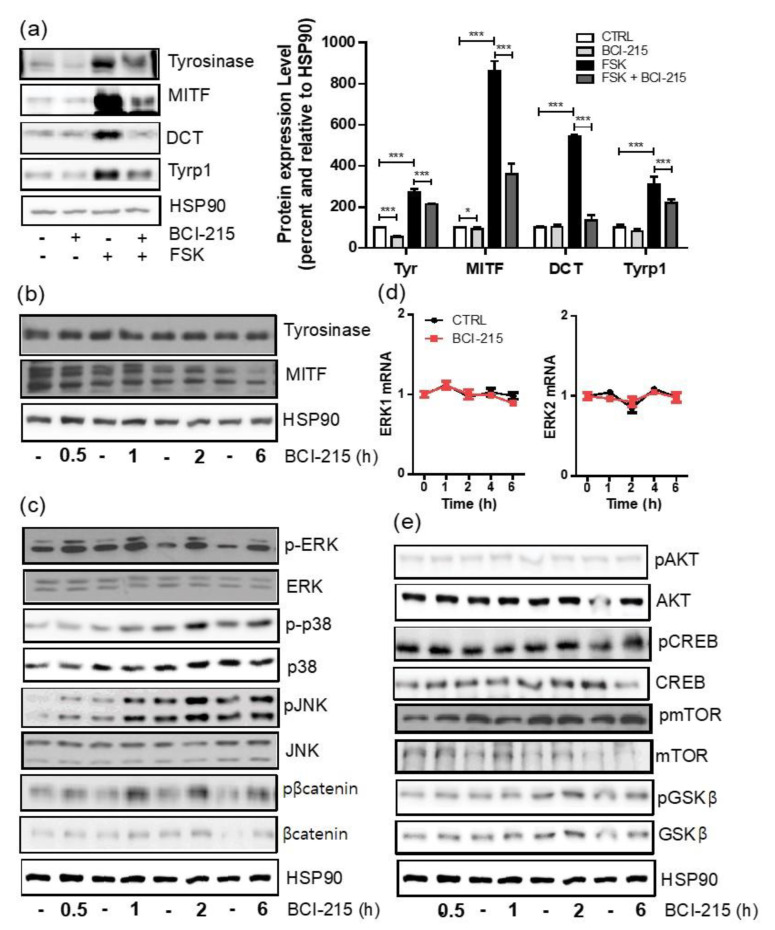
Effect of BCI-215 on the translation of MITF and its downstream genes and intracellular signaling expression. (**a**) Immunoblotting (*n* = 5) of MITF, Tyrp1, tyrosinase, and DCT 48 h after a treatment with 1 μM BCI-215 in a basal or FSK-stimulated condition. Quantitative data in the right panel. (**b**) Immunoblotting (*n* = 5) of MITF and tyrosinase up to 6 h after a treatment with BCI-215. (**c**) Protein expression (*n* = 10) of intracellular signaling pathways for the MAPKs, including total or phosphorylated forms of ERK, JNK, p38, and β-catenin after a 1 μM BCI-215 treatment at an indicated time. (**d**) Relative mRNA expression of ERK1/2 up to 6 h after exposure to 1 µM BCI-215. (**e**) The protein levels (*n* = 5) of the total or phosphorylated forms of mTOR, CREB, GSK-β, and AKT. – means control for each time point. * *p* < 0.05, *** *p* < 0.001 vs. controls.

We then investigated whether the decrease in melanogenesis by BCI-215 was associated with the downregulation of the protein levels of MITF and melanogenesis-involved genes instead. Reflecting the reduced melanin content at 72 h after the BCI-215 treatment (Figure 2d), immunoblotting showed that MITF and the tyrosinase protein levels in both the basal and FSK-stimulated condition of the Mel-ab cells were strongly downregulated 48 h after the BCI-215 treatment (Figure 4a). Similarly, the Tyrp1 and DCT protein levels in the FSK-stimulated Mel-ab cells were also downregulated by the BCI-215 treatment compared with the vehicle-treated control (Figure 4a). As the downregulation of the MITF mRNA levels by BCI-215 in a 6 h frame was smaller (Figure 3a), these results indicated that BCI-215 mainly regulated the protein turnover rather than the transcription of MITF. Moreover, the protein level of MITF, but not tyrosinase, was acutely downregulated within 6 h after the BCI-215 treatment (Figure 4b). 

### 2.4. BCI-215 Induces the Activation of All MAPKs, Including ERK, JNK, and p38 in Mel-Ab Cells

As MAPK activation was previously reported to induce the degradation of the MITF protein and a subsequent decrease in melanogenesis, we further investigated whether MAPKs or other intracellular signaling pathways were altered by a BCI-215 treatment [15]. The phosphorylation of ERK and JNK was immediately upregulated and maintained for at least 6 h after the BCI-215 treatment (Figure 4c). On the other hand, the protein levels of ERK and JNK as well as the mRNA levels of ERK1/2 after an exposure to 1 µM BCI-215 remained unchanged (Figure 4c,d). The treatment of the Mel-ab cells with BCI-215 also increased the phosphorylation of p38, but to a lesser extent compared with ERK and JNK (Figure 4c). Moreover, the phosphorylation of β-catenin in the Mel-ab cells was significantly increased by the BCI-215 treatment compared with the vehicle-treated Mel-ab cells (Figure 4c). The protein levels of the phosphorylated forms of mTOR, CREB, AKT, and GSK-β were not altered at the indicated time points (Figure 4e). Taken together, and consistent with its phosphatase inhibition functions, BCI-215 inhibited melanogenesis by activating multiple MAPKs, including ERK, JNK, and p38; the degree of activation was greater in ERK. Additionally, the phosphorylation of the β-catenin pathway and an early time reduction for the MITF transcription could contribute to the anti-melanogenic effect of BCI-215.

### 2.5. BCI-215 Effectively Reduces Melanogenesis in Human Keratinocyte-Cocultured Melanocytes and Ex Vivo Human Skin 

**Figure 5:**
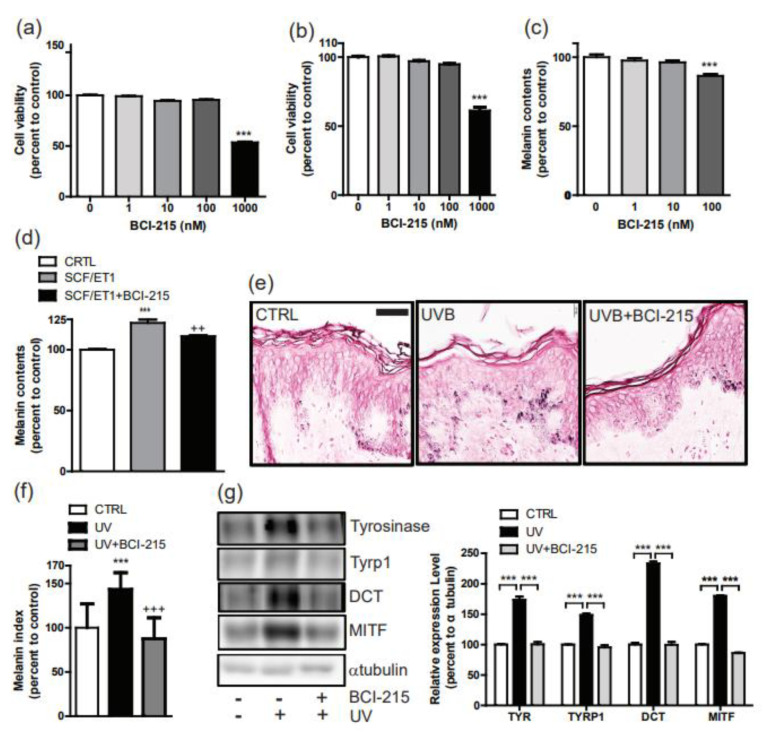
Effect of BCI-215 on melanogenesis in human keratinocyte-cocultured melanocytes and ex vivo human skin. Cellular viability was measured using a WST assay in (**a**) NHM and (**b**) NHK treated with 1–1000 nM of BCI-215 for 72 h. (**c**) The effect of a 100 nM dose of BCI-215 on the melanin content in NHM for the 72 h treatment. (**d**) The effect of BCI-215 on the melanin content in NHM cocultured with NHK stimulated by SCF and ET-1. ++ *p* < 0.01 vs. SCF and ET-1 treated NHM and NHK coculture. (**e**) Fontana–Masson staining of ex vivo human cultured skin tissue treated with a 200 mJ/cm^2^ dose of UVB with or without the BCI-215 treatment. (**f**) The fraction of the area showing positive Fontana–Masson staining was calculated to determine the melanin index. +++ *p* < 0.001 vs. UV treated ex vivo skin tissue. (**g**) Immunoblotting (*n* = 5) of the ex vivo human cultured skin tissue treated with UVB with or without a BCI-215 treatment with MITF, Tyrp1, tyrosinase, and DCT antibodies. Quantitative data in the right panel. – means vehicle treated control. *** *p* < 0.001 vs. controls.

BCI-215 at 1000 nM showed a cytotoxic effect on NHM and NHK in a WST cell proliferation assay (Figure 5a,b); a 100 nM dose of BCI-215 for 72 h decreased the melanin content in the NHMs (Figure 5c). Furthermore, BCI-215 significantly reduced the melanin content in the NHM and NHK cocultures, which were stimulated by SCF plus ET-1 (Figure 5d).

Using Fontana–Masson staining of ex vivo human cultured skin tissue, we found that a 200 mJ/cm^2^ dose of UVB increased the number of Fontana–Masson (+) melanosomes whereas a BCI-215 treatment + UVB resulted in a remarkable reduction in the number of melanosomes (Figure 5e). The fraction of the area showing positive Fontana–Masson staining was calculated to determine the melanin index; specimens treated with UVB and BCI-215 + UVB were thereby compared. The melanin index measurements revealed a significant increase after UV exposure, but a marked decrease after the BCI-215 treatment of the UVB-irradiated specimens (Figure 5f). Intriguingly, Western blotting with the lysed skin tissues at 96 h after the UVB and BCI-215 treatment showed that tyrosinase, Tyrp1, and DCT significantly decreased. There was also a modest, but significant, reduction in MITF protein compared with the vehicle + UVB (Figure 5g). 

## 3. Discussion 

For the treatment of hyperpigmentary disorders, a variety of whitening agents have been investigated and developed to date. Among them, hydroquinone and kojic acid are still widely used as skin bleaching topical agents because they act as tyrosine inhibitors; there are few other effective alternatives. However, hydroquinone cream can cause undesired hypopigmentation and also shows cytotoxic effects at high concentrations [16]. Furthermore, kojic acid and its derivatives may be unstable in cosmetic formulations or at high temperatures, occasionally causing contact dermatitis or even paradoxical pigmentation [17,18]. Other treatment options for hyperpigmentation such as antioxidants, laser treatments, retinoids, and chemical peeling are not suitable for general applications due to their limited effects and high costs [19,20]. Hence, efforts to discover effective, but safe, whitening agents are ongoing. Vitiligo and post-inflammatory hypopigmented skin lesions can be improved by melanogenic stimulators, but few such agents have been developed to date other than a few phototoxic plants or chemicals [21,22]. Therapeutic regimens of vitiligo include a 308 nm excimer laser, narrow-band UVB, oral or topical steroids, topical calcineurin inhibitors, and topical Janus kinase inhibitors [23,24,25]. Acquired hypopigmented lesions such as idiopathic guttate hypomelanosis or post-inflammatory hypopigmentation have been treated with cryotherapy, excimer lasers, a topical 0.1% tretinoin treatment, and narrow-band UVB [22]. However, the treatment of vitiligo or hypopigmented lesions is often unsatisfactory and inconsistent [20,26].

We designed a novel approach to explore an anti-melanogenic agent through protein kinase modulation rather than tyrosinase inhibitors. Protein kinases are enzymes that control the activity of specific proteins by attaching a phosphate to either the serine, threonine, or tyrosine residue on those proteins. MAPKs are a family of protein kinases; three major members are ERK, JNK, and p38 [27]. The ERK signaling pathway plays an important role in various cellular responses such as cell proliferation, survival, and differentiation [9,11,14]. Moreover, ERK 1/2 are both involved in complex signaling during melanin synthesis [11,12,13,15]. Previous studies have revealed that delayed ERK activation downregulates melanin synthesis via the degradation of the MITF protein [24,25]. 

In this context, and with respect to the effective regulation of melanogenesis, the MAPK phosphatases belonging to the family of DUSPs, which are specific inhibitors of MAPKs, became a potentially useful avenue of inquiry. The DUSPs exert their function by reversing the phosphorylation of MAPKs, resulting in their deactivation. Approximately 16 types of DUSP to date have demonstrated a dephosphorylating activity toward MAPKs in vitro [13]. DUSPs have also shown different patterns of substrate specificity, tissue expression, and subcellular localization according to the cell types [13]. In keratinocytes, previous studies have shown that the expression of DUSP2 and DUSP5 changed in response to an adalimumab treatment in psoriasis patients whereas arsenic, a human carcinogen that can cause squamous cell carcinomas (SCCs), could inhibit the expression of DUSP2 or DUSP14 [28,29]. During photodynamic treatments of human SCCs with hypericin, the apoptosis of the SCC cells activated DUSP1 via H3 histone modifications [30]. A functional impairment in DUSP1 was also found to be associated with a monocyte cellular dysfunction induced by low density lipoprotein in hypercholesterolemia patients [31]. A decreased DUSP1 expression showed an association with diabetes-associated cardiac hypertrophy [32]. 

The role of DUSPs in melanocytes regarding melanogenesis remains limited; to the best of our knowledge, only one article to date has shown that the mechanism of sphingosylphosphorylcholine-induced hypopigmentation involved a member of this family (DUSP6) in melanocytes [33]. Those authors reported that DUSP6 was upregulated in human melanocytes and could dephosphorylate ERK1/2 in these cells [33]. A few DUSPs are capable of inactivating several MAPKs whereas others act more specifically toward one type of MAPK. DUSP6 is known to be 100-fold more active toward ERK2 than either JNK or p38 [14,34]. On the other hand, DUSP1 can inactivate both ERK 1/2 and also JNK and p38 to a different extent depending on the cell type [35]. However, the role of DUSP1 has not yet been investigated in melanogenesis [13,35]. 

We previously demonstrated that MAPK pathway activation inhibited melanin accumulation via a decreased MITF [12]. Thus, we thought that an efficient modulation of MAPK could be a good strategy for interventions aiming to regulate pigmentation. It led us to explore other MAPK activators, but these factors are often only transiently activated. Thus, the inhibition of phosphatases for regulating the MAPK function seemed to be a more efficient approach. We observed that BCI-215, a DUSP1/6 inhibitor in B16F10 cells, reduced the melanin content efficiently in B16F10 melanoma cells. (E)-2-benzylidene-3-(cyclohexylamino)-2,3-dihydro-1H-inden-1-one (BCI), as seen in Figure 1a, was originally identified as an allosteric inhibitor of DUSP6/Mkp3 using zebrafish [36]. Analogs of BCI were efficiently prepared following a convergent synthetic method for BCI comprising the aldol condensation of dihydroindenones with benzaldehydes and the subsequent SN2 addition of amines. 5-Bromo analog BCI-215, (E)-2-Benzylidene-5-bromo-3-(cyclohexylamino)-2,3-dihydro-1H-inden-1-one, was reported to have a similar activity to its parent compound BCI with less toxicity, as seen Figure 1b [37]. BCI-215 was further investigated as a tumor cell selective inhibitor of MAPK phosphatases [38].

In this study, we observed that BCI-215 suppressed melanin synthesis in both cAMP-stimulated melanocytes and UV-stimulated ex vivo cultured human skin. BCI-215 treatments decreased the melanin synthesis mainly through ERK phosphorylation, but the effects on JNK and p38 phosphorylation were also noted in this context. As a DUSP6 inhibitor is known to specifically activate ERK, we speculated that a DUSP1 inhibitor may be responsible for JNK and p38 phosphorylation. In other words, the modulation of JNK or p38 by DUSP1 could additionally enhance the anti-melanogenesis effect of DUSP6 via sustained ERK activation up to 6 h after the BCI-215 treatment. Due to the dynamic interaction and feedback that occurs between the MAPK cascades, inactivating several MAPKs at once can induce long-lasting hypopigmentation effects. Moreover, we found that BCI-215 exposure led to a reduction in MITF transcription at an early time point, possibly via β-catenin phosphorylation, thus further suppressing melanogenesis as β-catenin signaling also plays a significant role in the upregulation of MITF and melanogenesis [39,40]. To acquire more effective whitening agents, there have been industrial or laboratory trends of mixing several chemicals with different working mechanisms. From that point of view, BCI-215 or its analogs may have potential advantages.

## 4. Materials and Methods

### 4.1. Compounds and Cell Culture Reagents

BCI-215, a chemical produced from BCI by medicinal chemistry [36,37,38], was purchased from MCE (MedChemExpress, Monmouth Junction, NJ, USA) and prepared as a solution in DMSO. Kojic acid, cholera toxin (CT), and 12-O-tetradecanoylphorbol-13-acetate (TPA) were purchased from Sigma-Aldrich Co. (St. Louis, MO, USA). Dulbecco’s Modified Eagle Medium (DMEM), Dulbecco’s phosphate-buffered saline (DPBS), and fetal bovine serum (FBS) were purchased from WelGENE (Daegu, South Korea). Medium 254, EpiLife™ Medium, Human Melanocyte Growth Supplement (HMGS), Human Keratinocyte Growth Supplement (HKGS), Antibiotic-Antimycotic (AA), and trypsin-EDTA were purchased from Gibco (Grand Island, NY, USA). Medium 254 (Gibco) was obtained from Cascade Biologics (Portland, OR, USA).

### 4.2. Cell Lines and Cell Culture

B16F10 murine melanoma cells (The Korean Cell Line Bank, Seoul, Korea) were cultured in DMEM supplemented with 10% FBS and 1% Antibiotic-Antimycotic solution (AA) (Thermo Scientific, Rockford, IL). Mel-ab cells from a mouse-derived spontaneously immortalized melanocyte cell line were obtained from The Korean Cell Line Bank (KCLB, Seoul, South Korea), gifted from Amorepacific Corp. (Seoul, Korea), and maintained in DMEM supplemented with 10% FBS, 1% AA, 100 nM TPA, and 1 nM CT. All cells were routinely maintained at 37 °C in a humidified environment of 5% CO2. Normal human melanocytes (NHM) (Invitrogen, Carlsbad, CA, USA) were cultured in Medium 254 supplemented with HMGS and 1% AA. Normal human keratinocytes (NHK) (KCLB, Seoul, South Korea) were cultured in EpiLife™ Medium supplemented with HKGS and 1% AA. The coculture of NHM and NHK was performed by seeding a 6-well cell culture plate at a density of 3.0 × 10^5^ cells/well of NHM in Medium 254. One day after, NHK in EpiLife™ Medium was added at 3.0 × 10^5^ cells/well to each well at a ratio of 1:1 NHM:NHK. After 24 h, the media were changed with the EpiLife™ Medium and stimulated with 10 ng/mL stem cell factor (SCF) and 0.1 nM endothelin-1 (ET-1). All cells were routinely maintained at 37 °C in a humidified environment of 5% CO2.

### 4.3. Quantitative Real-Time PCR

The total cellular RNA was isolated from the Mel-ab cells using a FavorPrepTM Total RNA Purification Mini Kit. Single-stranded cDNA was synthesized from RNA using a RevertAid First Strand cDNA Synthesis Kit in accordance with the manufacturer’s instructions (Thermo Scientific, Rockford, IL). A quantitative real-time PCR was performed using a LightCycler^®^ 480II machine with a Fast SYBRTM Green Master Mix value. The cDNAs were amplified using the primers listed in Table 1.

### 4.4. Western Blotting and Antibodies

For the Western blotting, the cells were washed once with cold PBS and lysed in a protein lysis buffer (1% SDS in 10 mM Tris and 5 mM EDTA, pH 7.4) followed by incubation at 98 °C for 10 min. The extracted protein samples from the cells or human skin tissues were separated by 8% SDS-polyacrylamide gel electrophoresis and blotted onto nitrocellulose membranes (GE Healthcare Life Science, Amersham, Marlborough, MA, USA), blocked with Tris-buffered saline containing 0.1% Tween 20 and 5% BSA. The membranes were then subjected to immunoblotting with the antibodies listed below. 

Tyrosinase, Tyrp1, and DCT antibodies were purchased from Abcam (Cambridge, UK). The MITF was from Cell Signaling Technology (Danvers, MA, USA). The antibodies against the total and phosphor form of AKT, ERK, β-catenin, CREB, GSK-β, p38 MAPK, SAPK/JNK, and mTOR were purchased form Cell Signaling Technology. Antibodies against HSP 90α/β (Santa Cruz, Dallas, TX, USA) or α-tubulin (Gentex, Holland, MI) were used as an internal loading control.

### 4.5. Cell Treatments and Cytotoxicity

Briefly, B16F10 cells and Mel-ab cells were seeded into 6-well cell culture plates at a density of 1.5 × 10^5^ cells/well in DMEM supplemented with 10% FBS and 1% AA. After 24 h, the media were changed and supplemented with 100 ng/mL α-melanocyte-stimulating hormone (MSH) for 1 h. The Mel-ab cells were seeded into 6-well cell culture plates at a density of 3.0 × 10^5^ cells/well in DMEM containing 10% FBS and 1% AA without CT and TPA. After 24 h, the media were changed and supplemented with 10 µM FSK for 1 h. The NHM cells were seeded into 6-well cell culture plates at a density of 6.0 × 105 cells/well in the M254 medium with HMGS and 1% AA. The cells were then incubated for 72 h. 

The cytotoxic effects of BCI-215 in Mel-ab, NHM, and NHK were evaluated using a Ez-Cytox Cell Viability Assay Kit (Dogen-Bio Co., Ltd., Seoul, Korea) in accordance with the manufacturer’s instructions.

### 4.6. Melanin Content Measurements

Prior to measuring the melanin content, the cells were observed under a phase contrast microscope (Olympus, Tokyo, Japan) and photographed. The cells were then dissolved in 550 µL of 1 N NaOH at 100 °C for 30 min and centrifuged at 13,000 rpm for 5 min. The absorbance of the supernatants was measured at 405 nm using a microplate reader. The intracellular melanin content was presented as a percentage relative to the untreated cell controls.

### 4.7. Cellular Tyrosinase Activity 

The cellular (indirect) tyrosinase activity level was evaluated by measuring the rate of dopachrome formation from L-DOPA (L-3,4-dihydroxyphenylalanine). After incubation with BCI-215, the cells were washed in ice-cold PBS and lysed in a tyrosinase lysis buffer (phosphate buffer, pH 6.8, containing 1% Triton X-100) with repeated freeze/thaw cycles. The lysates were clarified by centrifugation at 15,000 rpm at 4 °C for 10 min. After quantifying the protein levels of the lysate and adjusting the protein concentrations with a lysis buffer, 90 µL of the supernatant mixed with 10 µL of 10 mM L-DOPA in a tyrosinase lysis buffer was incubated at 37 °C. The tyrosinase activity was measured by reading the absorbance at 475 nm every 10 min for at least 1 h using a microplate reader. A total of 200 μM of kojic acid was used as a positive control. 

### 4.8. Ex Vivo Human Skin Culture and Melanin Index of Human Skin

Human skin tissues were obtained with informed consent from patients who had undergone neck or abdominal reduction surgery and were used in accordance with the guidelines of our Institutional Review Board (2020-0091). The skin samples were washed with 70% to 100% ethanol and kept moist in a Petri dish with PBS. To culture these tissues, the skin was first cut into 2 cm^2^ sections and then placed on the bottom of a transwell with the epidermis facing up in order to be exposed to the air. The explants were incubated in a humidified environment at 37 °C and in 5% CO2. The culture medium consisted of DMEM supplemented with 10% FBS and 10% AA and was replaced each day. For UV radiation-stimulated melanogenesis, the skin explants were irradiated with 200 mJ/cm^2^ UVB every 48 h for 30 s and treated with 10 µM BCI-215 in 30% propylene glycol and 70% EtOH for 96 h. The skin tissue was then harvested and fixed in 10% formalin prior to embedding in paraffin. An image analysis was used to determine the relative band densities using Image J software (version 1.53K, National Institutes of Health, Bethesda, MA, USA).

Paraffin-embedded human skin tissues were cut into 6 μm-thick sections and stained using a Fontana–Masson kit (ID labs, London, ON, Canada) in accordance with the manufacturer’s instructions. The melanin index was determined by measuring the percentage of the stained area in relation to the total tissue area using ImageJ software (version 1.53K, National Institutes of Health).

### 4.9. Statistics

The data in this study are presented as mean values ± standard error of the mean (SEM); the statistical significance was determined using an unpaired Student’s *t*-test with GraphPad Prism software (version 5, San Diego, CA USA). *p* values < 0.05, < 0.01, and < 0.001 were considered to be statistically significant and were represented by *, **, and ***, respectively.

## 5. Conclusions

In conclusion, we showed that a BCI-215 inhibitor effectively downregulated melanogenesis in melanocytes and human ex vivo skin, mainly through the ERK/MITF pathway and partially via JNK, p38, and β-catenin signaling, indicating a useful therapeutic strategy for UV-associated skin pigmentary disorders. These results also suggested that BCI-215 and its analogs, using phosphatase modulation for regulating multiple cellular signaling events, are promising for developing effective regulators of melanogenesis, either through enhancement or suppression via the pharmacological modulation of DUSP-related pathways.

## Figures and Tables

**Table 1:** List of primers used for quantitative real-time PCR.

Name	Forward	Reverse
GAPDH	CATCACTGCCACCCAGAAGACTG	ATGCCAGTGAGTTCCCGTTCAG
MITF	GGGATGCCTTGTTTATGGTG	CACCGCAGACCACTTAGTCC
Tyrosinase	TTATGCGATGGAACACCTGA	GAGCGGTATGAAAGGAACCA
Tyrp1	CCCCTAGCCTATATCTCCCT	TACCATCGTGGGGATAATGG
DCT	CTTTGCAACCGGGAAGAACG	CCGACTAATCAGCGTTGGGT
OCA2	ATAGTGAGCAGGGAGGCTGT	ACTGATGGGCCAGCAAAAGA
ERK1	CCTGCTGGACCGGATGTTA	TGAGCCAGCCTTCCTCTAC
ERK2	GGAGCAGTATTATGACCCAAGTGA	TCGTCCACTCCATGTCAAACT

## Data Availability

The datasets generated during and/or analyzed during the current study are available from the corresponding author on reasonable request.
